# Annotations

**Published:** 1902-06-07

**Authors:** 


### ANNOTATIONS.

### The Law and Unqualified Practice.

COLONIAL law makers have at least the virtue of going straight to the point, whereas with us it too often happens that the efforts made to avoid treading upon anybody's toes render our legislation useless for any good purpose. Good illustrations of the sort of laws with which we have to be content in England are the Medical Acts and the Midwives Bill, which is now before Parliament, both of which, starting on the pretence of protecting the public from unqualified practice, end with impotent provisions for merely penalising the assumption of certain titles. In New Zealand the harm done by unqualified practice is quite as apparent as it is here, and the Minister of Public Health introduced into the New Zealand Parliament, last session, a new Medical Bill, some of the provisions in which are worthy of note. One clause provides that "no person shall practise medicine, surgery, or midwifery, or the administration of any medical treatment," unless he is on the Register; while another provides that every person commits an offence, and is liable to a penalty up to £50 or imprisonment up to 12 months, "who, not being duly registered, takes or uses the name or title of physician, doctor of medicine, licentiate, bachelor, or master in medicine or surgery, surgeon, general practitioner, medical practitioner, medical specialist, medical dispenser, apothecary, aurist, ophthalmist, medico-herbalist, medico-electrician, or any of these or the like titles with any added words, variations, or designations, or any title, style, or description, directly or indirectly implying" that he is possessed of any degree or license, or of the skill necessary to practise medicine, surgery, or midwifery. There is something like a Bill! There is no question here of "wilfully and falsely" using these titles. If a man uses them at all he is liable. Evidently the people in New Zealand who wish to put a stop to unqualified practice frame their measures so as to meet the end in view. We, however, are of softer fibre; we profess to aim at the quack, but aim a little on one side lest the poor fellow should be hurt or his "liberty" to do as he likes be interfered with.

### The Chronic Student.

THERE is no doubt that the "chronic" deserves much that is said against him. He tries his teachers' patience and strains his guardian's purse by his repeated failures, and is a distinctly evil influence in any school. Sometimes he is what one may call a "shocking example," thus perhaps serving a purpose by pointing a moral, and sometimes he is merely lazy and deserves expulsion. But there are other "chronics" who must be placed in quite a different category—good, steady fellows, always interested, always ready to turn their hands to anything, useful in the wards, often the favourites of the sisters and the nurses, in a certain sense the favourites of the professors, seeing that they can be trusted and relied upon, clever with their fingers, men who are good in all the practical departments, and yet fail before the ordeal of the examination. The harm of having such men about a school is that they give a sanction to non-success, and by the estimation in which they are sometimes held, take away from the disgrace of failure at examinations. Yet it is not easy to see what can be done with them, for they may make good practical practitioners in the end, and by their long familiarity with ward work develop surgical and other instincts which may prove better guides in practice than much of the book knowledge of those clever people who face the examiners with such a light heart and floor them with such ready quotations. The suggestion made last week by Mr. Ball to the General Medical Council that after each successive failure to pass an examination an increased length of reference should be demanded, would no doubt go far to diminish the evil. It would at any rate lead students to think much more seriously of their examinations, and put a stop to the great evil of so many of them "going in" half prepared on the chance of scraping through. But nothing will avail to bring all students to the same level. Examinations are doubtless the best means at our disposal by which to judge of fitness. They are, however, but a rough and ready method even at the best, and it is important that in our striving for uniformity we take care not to do injustice to that group of slow learners who form the better class of "chronics"—men who in the end often make very good practitioners.

### The Advantages of a Broken Head.

PRACTICALLY all cases in which people are so smitten with sudden illness as to become unconscious in the street fall into the hands of the police. Under such circumstances lucky is the invalid if in falling he cuts his head, and bleeds with sufficient profusion to be classed as an "accident," for then will he be taken to a hospital. Otherwise the chances are that he will be treated as a "drunk," especially if on first feeling ill he has taken ever so small a dose of brandy, and thus produced that "breath smelling of spirits" which has condemned so many a man to death in a police cell. It is a curious contrast to see the riotous brawler with the broken head being carefully attended to by skilful doctors and kindly nurses, while the quiet and harmless drinker whose only offence is that he tumbles down and lies in everybody's way, is thrown to sleep it off upon the plank bed of a police station. But the serious part of the business is that the unfortunate who being attacked with sudden illness—ingravescent apoplexy or the like—becomes unconscious in the street, is classed with the "drunk and incapables"—unless, lucky man, he cuts his head, for then he may be taken to hospital. Knowing, as we do the frightful nuisance caused to the sick by the intrusion of drunken people into hospital wards at all times of the night, we would be the last to suggest that every unconscious person picked up by the police should be taken to a hospital. What we do urge is that every such person taken to the police station should be seen by a medical practitioner, and should under no circumstances be shut up to "sleep it off," or left in the hands of unskilled persons. We are not quite satisfied that the arrangements with the divisional surgeons in regard to "calling up" at night are altogether what they should be. Whatever the expense something should be done to prevent the danger of "death in the cells" which at present hangs over those who happen to be attacked in the streets with illness simulating drunkenness.

